# Early-life milk production traits predict lifetime milk yield and support replacement decisions in Holstein, Jersey and Crossbred dairy cows under subtropical conditions

**DOI:** 10.1016/j.vas.2026.100841

**Published:** 2026-08-27

**Authors:** Onke Tshona, Ziyanda Mpetile, Oluwakamisi Festus Akinmoladun

**Affiliations:** a Department of Animal and Pasture Science, Faculty of Science and Agriculture, University of Fort Hare, Private Bag X1314, Alice 5700 Eastern Cape, South Africa; b Discipline of Agriculture and Food Technology, School of Agriculture, Geography, Environment, Oceans and Natural Science, The University of the South Pacific, Samoa Campus, Apia, Samoa

**Keywords:** Dairy cattle, Lifetime milk yield, First-lactation milk yield, Replacement decisions, Receiver operating characteristic analysis, Subtropical production systems

## Abstract

Improving lifetime milk yield through the early identification of superior replacement animals is a key objective of sustainable dairy production systems. This study investigated the potential of first-lactation performance indicators to predict lifetime milk yield in dairy cattle managed under subtropical conditions. Historical production records from 349 Holstein, Jersey and crossbred dairy cows maintained at a commercial dairy farm were analysed using linear mixed models, multiple regression and receiver operating characteristic analysis. Estimated lifetime milk yield (ELMY) was calculated from average daily milk yield and lactation length across recorded lactations. Breed, parity, and their interaction significantly influenced several milk production traits (P < 0.05). Holstein cows exhibited the highest (P < 0.05) milk production traits, while milk production increased with advancing parity. Breed-related differences in milk production were evident across parities, with more pronounced variation among breeds at later parities (P_4_–P_5_) for selected traits. First-lactation daily milk yield (FDMY) was strongly associated with ELMY (r = 0.849, P < 0.001) and emerged as the most important predictor of lifetime milk yield. Final prediction model incorporating FDMY and breed explained 74.7% of the variation in ELMY (adjusted R² = 0.731). Cows classified as high lifetime producers had significantly greater FDMY than medium- and low-producing cows (P < 0.001). Binary logistic regression showed that first-lactation daily milk yield independently predicted high estimated lifetime milk-yield status after adjustment for breed (OR = 2.14, 95% CI: 1.45–3.17; *P* < 0.001), with an overall classification accuracy of 90.6%. Receiver operating characteristic analysis demonstrated excellent discriminatory ability of FDMY (AUC = 0.917 ± 0.036, P < 0.001), with an optimal threshold of 16.0 kg/day providing 76.2% sensitivity and 93.7% specificity. These findings identify first-lactation milk yield as a promising early-life indicator of subsequent estimated lifetime milk yield.

## 1 Introduction

Improving lifetime milk yield is a central objective of modern dairy production systems because it directly influences farm profitability, resource-use efficiency, replacement costs, and environmental sustainability. As the dairy industry faces increasing pressure to produce more milk with fewer resources, maximising the productive lifespan and efficiency of individual cows has become a major focus of breeding and management programmes (De Vries & Marcondes, 2020; Clasen et al., 2024). Cows that combine high milk production with sustained longevity contribute disproportionately to herd profitability by spreading rearing and replacement costs over a greater quantity of lifetime milk output (Bieber et al., 2026). Consequently, identifying management and biological factors that influence lifetime milk yield has become increasingly important in both intensive and pasture-based dairy systems.

Milk production is a complex trait determined by interactions among genetic, physiological, environmental, and management factors (Davis, 2017; Erdoğan et al., 2024). Among these, breed and parity are consistently recognised as major sources of variation in dairy performance. Breed differences reflect decades of selection for specific production objectives and adaptation to diverse production environments. Holstein cattle have traditionally been selected for high milk volume and generally outperform most dairy breeds for milk yield, whereas Jersey cattle are recognised for superior milk solids concentration, feed efficiency, and adaptability under certain production conditions (Olthof et al., 2023). Crossbreeding has received increasing attention as a strategy to combine desirable characteristics of specialised breeds while exploiting heterosis in production, fitness, and longevity traits (Clasen et al., 2017; Ferris et al., 2018). Understanding breed-related variation in milk production traits remains essential for developing breeding and replacement strategies that optimise long-term herd performance.

Parity represents another important determinant of milk production performance. Milk yield generally increases from first lactation to middle parities as cows reach physiological maturity, attain greater body size, and achieve full mammary gland development (Xiong et al., 2018). Similarly, peak milk yield, lactation persistency, and lactation yield are influenced by parity-dependent changes in nutrient partitioning, endocrine regulation, and metabolic capacity (Lacková et al., 2022; Vrhel et al., 2024). Characterising parity effects is therefore necessary to understand patterns of productive performance across the lifespan of dairy cows and to accurately evaluate breed differences under commercial production conditions.

While understanding factors affecting current lactation performance is important, producers increasingly require decision-support tools that facilitate the early identification of animals with superior long-term productive potential. Replacement and culling decisions are among the most economically important management choices in dairy systems because they influence herd milk yield, longevity, and profitability for many years (Schlebusch et al., 2024). However, these decisions are often made before complete lifetime performance information becomes available. Consequently, there is growing interest in identifying early-life indicators that can reliably predict future milk yield and support evidence-based replacement strategies (Dallago et al., 2024).

Lifetime milk production is an integrated measure of productive efficiency because it captures an animal's cumulative contribution across successive lactations. Previous studies have reported favourable relationships between first-lactation performance and subsequent lifetime milk yield, suggesting that information collected early in life may provide valuable insight into future performance potential (Jenko et al., 2015; Siriak et al., 2022). Traits such as first-lactation milk yield, peak milk yield, lactation persistency, and lactation length have been proposed as potential predictors of lifetime milk yield because they reflect both genetic merit and physiological capacity for sustained milk production. Nevertheless, the strength and practical utility of these relationships may vary among breeds, management systems, and production environments, highlighting the need for system-specific evaluations. This variability across production systems underscores the importance of determining whether predictive relationships established elsewhere can be reliably applied under different genetic and environmental conditions.

Although previous studies have demonstrated positive associations between first-lactation milk yield and subsequent lifetime milk yield, most have focused on single breeds, particularly Holstein cattle, under temperate production systems. Consequently, there is limited understanding of whether these predictive relationships remain consistent across different dairy breeds managed under subtropical production environments. Moreover, relatively few studies have progressed beyond establishing statistical associations to develop practical decision-support approaches that assist producers in making early replacement and retention decisions. Addressing these gaps would strengthen the application of first-lactation performance records as management tools for improving long-term herd milk yield. These knowledge gaps are particularly relevant in South Africa, where multiple dairy breeds are managed under subtropical production conditions and breed-specific information is required to support evidence-based herd management decisions.

Despite the economic importance of dairy production in South Africa, evidence on the combined influence of breed and parity on milk production traits across Holstein, Jersey, and Holstein × Jersey crossbred cows remain scarce. In addition, the practical application of first-lactation performance indicators for guiding replacement decisions under subtropical production conditions has received little attention. Evaluating these relationships across multiple genetic groups and translating them into practical decision-support criteria may provide producers with a simple, objective approach for identifying cows with superior long-term productive potential early in life. Therefore, the objectives of this study were to: (i) quantify the effects of breed, parity, and their interaction on daily milk yield, peak milk yield, lactation persistency, lactation length, and lactation yield in Holstein, Jersey, and crossbred dairy cows; and (ii) evaluate the ability of first-lactation production traits to predict lifetime milk yield and identify practical thresholds that could support replacement decisions in subtropical dairy production systems. We hypothesised that milk production traits would differ among breeds and parities, and that first-lactation milk production characteristics, particularly daily milk yield, would predict lifetime milk yield.

## 2 Materials and methods

### 2.1 Study site and data source

This study used historical dairy production records from the University of Fort Hare Dairy Farm, a medium-to-large commercial dairy farm located in Alice, Eastern Cape Province, South Africa. The farm maintains Holstein, Jersey, and Holstein × Jersey crossbred dairy cattle under intensive management and routinely records milk production and reproductive performance data for herd management purposes. Production records spanning multiple lactations were extracted from the farm database and used to evaluate the effects of breed and parity on milk production traits and to assess the ability of first-lactation performance indicators to predict lifetime milk yield.

### 2.2 Data editing and study population

The extracted dataset contained information on cow identification number, breed, parity, daily milk yield, peak milk yield, lactation persistency, and lactation length. Data screening and quality control procedures were conducted prior to statistical analysis to ensure completeness and consistency of records.

For the evaluation of breed and parity effects on milk production traits and the prediction of lifetime milk yield, records were retained if breed identity and parity number were available, had complete first-lactation records for daily milk yield, peak milk yield, persistency, and lactation length, had at least three consecutive lactation records, and had complete production records for all included lactations were included to ensure adequate longitudinal production records for estimating lifetime milk productivity and to reduce the influence of incomplete production histories associated with cows having only one or two lactations. Records with missing breed information, missing parity information, incomplete production records, incomplete first-lactation records, discontinuous parity histories or fewer completed lactations were excluded. Following data editing, 349 cows comprising 156 Holstein, 92 Jersey, and 101 crossbred cows were retained for analysis.

### 2.3 Estimation of lactation and lifetime milk yield

Direct lactation milk yield records were unavailable in the farm database. Therefore, lactation yield for each lactation was estimated as the product of average daily milk yield and lactation length:

Lactation yield for each lactation was estimated from daily milk yield and lactation length as:

(1) $$LY_{i}=DMY_{i}\times LL_{i}$$

where: $LY_{i}$= lactation yield (kg) for the $i^{th}$lactation; $DMY_{i}$= average daily milk yield (kg/day) for the $i^{th}$lactation; $LL_{i}$= lactation length (days) for the $i^{th}$lactation.

Estimation of Lifetime milk yield (ELMY) was estimated as the cumulative milk production across all available consecutive lactations:

(2) $$ELMY=\;\sum_{i=1}^{n}LY_{i}$$

where: ELMY= estimated lifetime milk yield (kg); $LY_{i}$= estimated milk yield during the $i^{th}$lactation (kg); n= total number of completed lactations included for a cow; i= lactation number.

The milk production traits evaluated were daily milk yield (kg/day), peak milk yield (kg/day), lactation persistency (%), lactation length (days), and lactation yield (kg). Breed was classified into three categories (Holstein, Jersey, and Crossbred), while parity was classified into five levels corresponding to first through fifth lactations.

### 2.4 Statistical analyses

All statistical analyses were performed using IBM SPSS Statistics Version 20 (IBM Corp., Armonk, NY, USA). Statistical significance was declared at P < 0.05 unless otherwise stated.

Because repeated lactation records were available for individual cows across successive parities, observations were not independent. Therefore, linear mixed models (LMM) were fitted to evaluate the effects of breed, parity, and their interaction on daily milk yield, peak milk yield, lactation persistency, lactation length, and lactation yield while accounting for repeated measurements within cows.

*The model fitted was:*

$$Y_{ijkl}=\mu +B_{i}+P_{j}+{\left(BP\right)}_{ij}+C_{k}+\varepsilon_{ijkl}$$

where: $Y_{ijkl}$= observation of the milk production trait; μ= overall mean; $B_{i}$= fixed effect of the $i^{th}$breed (Holstein, Jersey, or Crossbred); $P_{j}$= fixed effect of the $j^{th}$parity (1–5); ${\left(BP\right)}_{ij}$= breed × parity interaction; $C_{k}$= random effect of the $k^{th}$cow; and $\varepsilon_{ijkl}$= residual error term.

Model parameters were estimated using restricted maximum likelihood (REML). The significance of fixed effects was assessed using Type III F-tests. Where significant breed or parity effects were detected (P < 0.05), estimated marginal means were compared using Bonferroni-adjusted pairwise comparisons. Results are presented as least-squares means ± standard error (SE). For traits exhibiting significant breed × parity interactions, interaction plots were generated to facilitate biological interpretation of breed-specific responses across successive lactations. Prior to model fitting, the assumptions of linear regression were assessed by examining residual plots for linearity and homoscedasticity, normal probability (Q–Q) plots of standardized residuals to evaluate residual normality, and variance inflation factors (VIFs) to assess multicollinearity among predictor variables. Model diagnostics indicated that the assumptions of linear regression were satisfactorily met.

Pearson correlation analysis was initially performed to evaluate relationships among candidate first-lactation predictor variables and estimated lifetime milk yield (ELMY). Candidate predictors included first-lactation daily milk yield (FDMY), peak milk yield (FPY), persistency (FPERS), and lactation length (FLL). Prior to correlation analysis, the normality of continuous variables was assessed using the Shapiro–Wilk test and visual inspection of Q–Q plots. Since the variables were approximately normally distributed, Pearson's correlation coefficients were calculated.

Prior to model fitting, multicollinearity among predictor variables was assessed using tolerance statistics and variance inflation factors (VIF). Variables with VIF values > 5 were considered to exhibit problematic collinearity. Because first-lactation peak milk yield showed strong collinearity with daily milk yield, it was excluded from subsequent regression analyses.

Multiple linear regression analysis was then used to evaluate the ability of first-lactation production traits to predict ELMY. Two modelling approaches were considered.

The initial model included first-lactation production traits only:

(3) ELMY=f(FDMY,FPY,FPERS,FLL)

A second model incorporated breed effect*:*

(4) ELMY=f(FDMY,FPERS,FLL,Breed)

where: FDMY= first-lactation daily milk yield; FPY= first-lactation peak milk yield; FPERS= first-lactation persistency; FLL= first-lactation length.

Breed was represented using dummy variables, with Holstein designated as the reference category. A hierarchical model selection procedure was adopted whereby predictors were retained based on statistical significance, biological relevance, absence of multicollinearity, and improvement in adjusted $R^{2}$. Variables that failed to contribute significantly to model performance were sequentially removed and reduced models were refitted.

Model performance was assessed using: i) coefficient of determination (R²), ii) adjusted coefficient of determination (Adjusted R²), iii) analysis of variance (ANOVA), iv) standard error of estimate, v) regression coefficients and vi) variance inflation factors (VIF).

To further evaluate whether the relationship between first-lactation milk yield and lifetime milk yield differed among breeds, breed-specific regression models were additionally fitted for Holstein, Jersey, and Crossbred cows separately.

Differences in first-lactation daily milk yield among estimated lifetime milk-yield classes (low, medium, and high producers) were evaluated using one-way analysis of variance (ANOVA), followed by Tukey's honestly significant difference (HSD) test for pairwise comparisons. This analysis was performed to describe differences in first-lactation daily milk yield across the three estimated lifetime productivity classes and to provide descriptive biological context for the predictive analyses.

To evaluate the ability of first-lactation daily milk yield to identify cows with high estimated lifetime productivity, binary logistic regression analysis was performed. Estimated lifetime milk yield was initially classified into low-, medium-, and high-productivity groups based on tertiles cut-off values. Tertile classification was selected to provide balanced group sizes for statistical comparison while maintaining clinically meaningful differentiation across the range of lifetime productivity. For the logistic regression analysis, cows in the low- and medium-productivity groups were combined to form the reference category (coded 0), whereas cows in the high-productivity group were coded as high producers (coded 1). First-lactation daily milk yield was entered as the primary predictor, while breed was included as an adjustment variable using dummy coding, with Holstein cows serving as the reference category. Regression coefficients (B), standard errors (SE), Wald χ² statistics, odds ratios (OR), and corresponding 95% confidence intervals (CI) were estimated. Model fit was evaluated using the Omnibus Test of Model Coefficients and the Hosmer–Lemeshow goodness-of-fit test. Model performance was further assessed using Nagelkerke's pseudo-R² together with classification accuracy, sensitivity, and specificity.

Receiver operating characteristic (ROC) curve analysis was subsequently performed to evaluate the discriminatory ability of first-lactation daily milk yield for identifying high estimated lifetime milk producers. The area under the ROC curve (AUC) was calculated as a measure of model discrimination, and the optimal decision threshold was determined using the Youden Index to maximise the combined sensitivity and specificity. Because the threshold was derived from the same dataset used to develop the prediction model, it is presented as a preliminary decision-support threshold requiring validation in independent populations.

The optimal first-lactation milk yield threshold was selected using the maximum Youden Index:

(5) J=Sensitivity+Specificity−1

## 3 Results

### 3.1 Breed and parity effects on milk production traits

The effects of breed, parity, and their interaction on milk production traits are presented in Table 1. Breed significantly influenced daily milk yield, peak milk yield, persistency, and lactation yield (P < 0.05), whereas no breed effect was observed for lactation length (P > 0.05). Parity similarly affected daily milk yield, peak milk yield, persistency, and lactation yield (P < 0.05) but had no significant effect on lactation length. Significant breed × parity interactions were detected for peak milk yield and persistency (P < 0.05), indicating that the response of these traits to increasing parity differed among breeds.

**Table 1:** Tests of fixed effects of breed, parity, and breed × parity interaction on milk production traits in 349 Holstein, Jersey, and crossbred dairy cows.

Source	DMY (kg)	PMY (kg)	Persistency (%)	LL (days)	ELMY (kg)
Breed	18.62⁎⁎	17.60⁎⁎	6.56*	0.99ns	11.71⁎⁎⁎
*p-value*	*<0.001*	*<0.001*	*0.002*	*0.375*	*<0.001*
Parity	21.07⁎⁎	87.01⁎⁎	5.81⁎⁎	0.93ns	9.15⁎⁎⁎
*p-value*	*<0.001*	*<0.001*	*<0.001*	*0.448*	*<0.001*
Breed x Parity	0.70ns	2.47*	2.16*	0.84ns	0.72ns
*p-value*	*0.690*	*<0.001*	*0.031*	*0.566*	*0.674*

⁎ : P < 0.05;.

⁎⁎ : P < 0.01;.

⁎⁎⁎ : P < 0.001; ns: not significant (P > 0.05); LL: lactation length; ELMY: lifetime milk yield; FDMY: daily milk yield; PMY: Peak milk yield.

The significant breed effects observed for milk production traits reflect inherent genetic differences among dairy breeds. Holstein cows exhibited the highest (P < 0.05) daily milk yield (17.13 ± 0.41 kg/day), peak milk yield (27.99 ± 0.59 kg/day), persistency (72.75 ± 0.65%), and lactation yield (5105.34 ± 139.35 kg), while Jersey cows recorded the lowest values for most production traits (Table 2).

**Table 2:** Estimated marginal means (± SE) of milk production traits according to breed and parity in 349 Holstein, Jersey, and crossbred dairy cows.

Variables	DMY (kg)	PMY (kg)	Persistency (%)	LL (days)	ELMY (kg)
Breed effects					
Holstein	17.13^a^	27.99^a^	72.75^a^	296.67	5105.34^a^
Jersey	13.16^c^	22.50^b^	70.23^a^^,^^b^	304.47	4055.44^c^
Crossbred	14.91^b^	26.61^a^	68.99^b^	295.67	4419.53^b^^,^^c^
*SEM*	*0.477*	*0.674*	*0.816*	*4.434*	*163.57*
*p-value*	*<0.001*	*<0.001*	*0.002*	*0.375*	*<0.001*
Parity effects					
1	13.38^b^	21.07^b^	72.63^a^	304.71	4073.77^a^
2	15.04^a^	24.71^c^	72.76^a^	298.61	4489.56^b^
3	15.89^c^	26.88^a^	70.02^b^	293.94	4662.51^b^
4	15.89^a^^,^^c^	27.47^a^	70.51^a^^,^^b^	301.33	4839.13^b^
5	15.14^a^^,^^c^	28.37^a^	67.39^a^^,^^b^^,^^c^	296.11	4568.89^b^
*SEM*	*0.477*	*0.443*	*0.863*	*5.285*	*183.97*
*p-value*	*<0.001*	*<0.001*	*<0.001*	*0.448*	*<0.001*

abc Means with different superscripts down the group are significantly different (P < 0.05); LL: lactation length; ELMY: lifetime milk yield; DMY: First-lactation Daily milk yield; PMY: Peak milk yield.

**Fig. 1:**
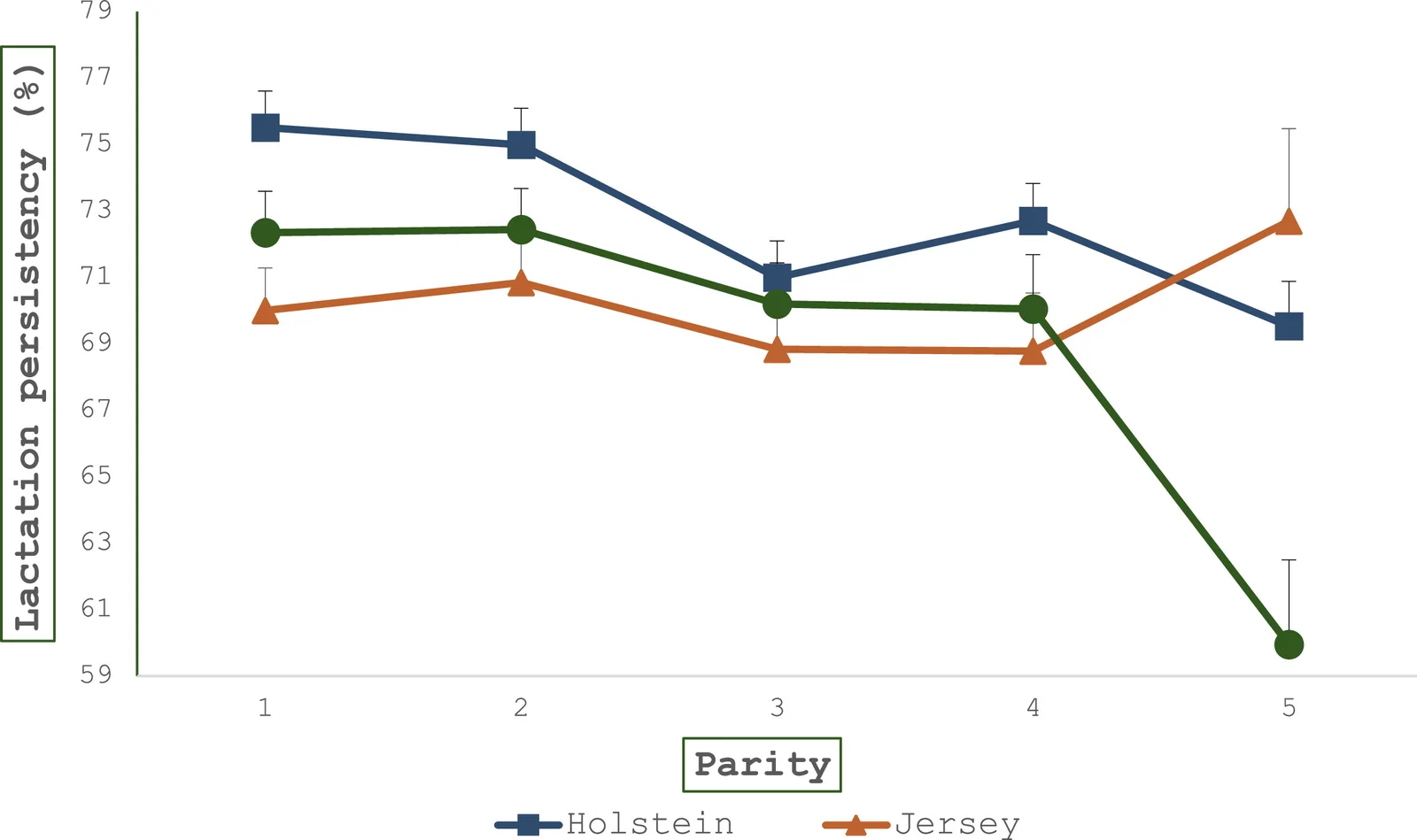
Interaction between breed and parity on estimated marginal means of lactation persistency in dairy cows. Error bars represent standard errors.

**Fig. 2:**
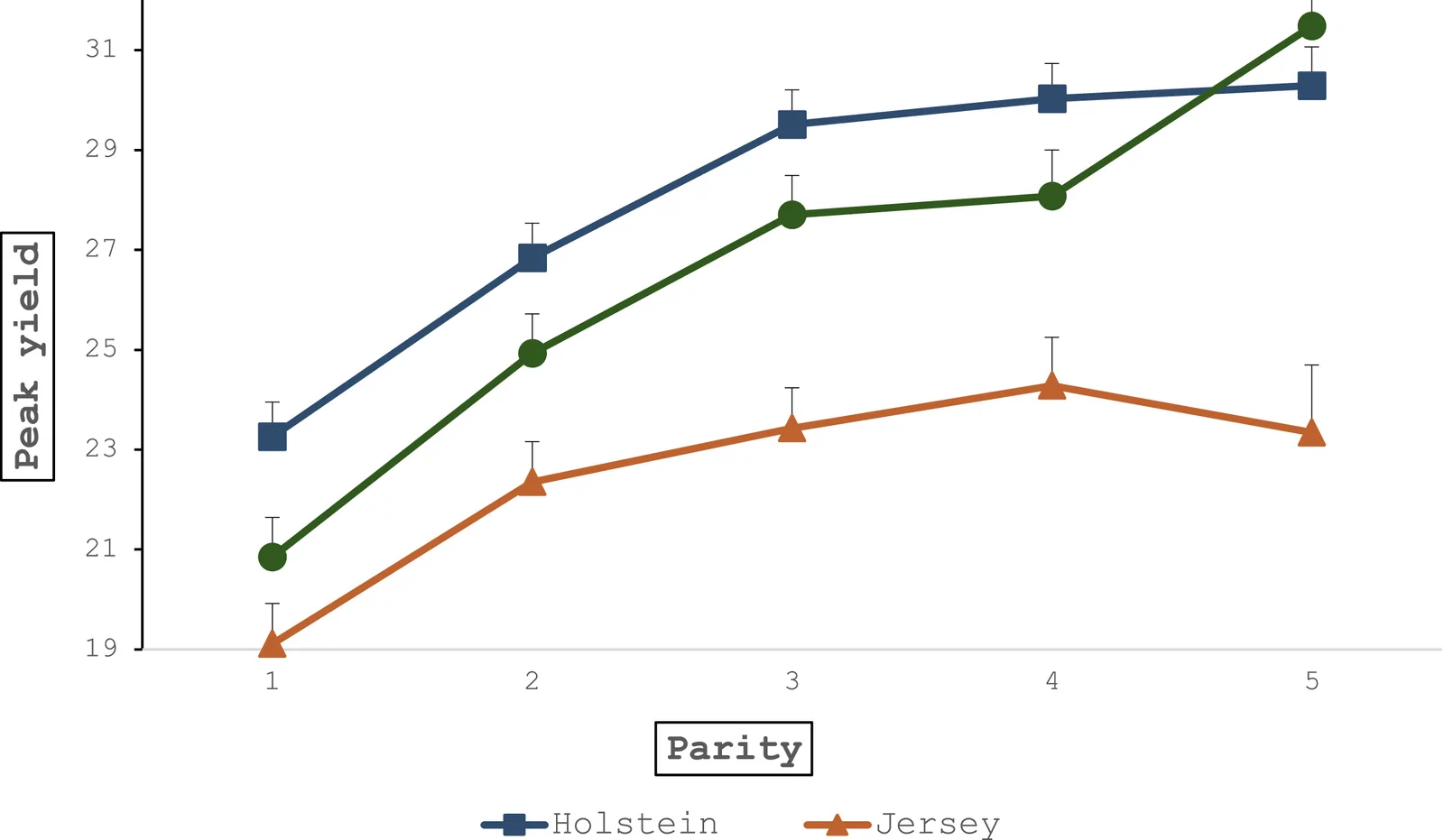
Interaction between breed and parity on estimated marginal means of peak milk yield in dairy cows. Error bars represent standard errors.

Parity exerted a pronounced influence on milk production traits. Daily milk yield increased (P < 0.05) from 13.38 ± 0.32 kg/day in first-parity cows to 15.89 ± 0.37 kg/day in fourth parity before stabilising thereafter. Similarly, peak milk yield increased progressively (P < 0.05) from 21.07 ± 0.44 kg/day in first parity to 28.37 ± 0.67 kg/day in fifth parity. Lactation yield followed a similar pattern, increasing by approximately 19% between the first and fourth parities. In contrast, lactation length was not significantly affected by breed or parity (P > 0.05), with values ranging between 294 and 305 days across treatment groups. The interaction between breed and parity significantly influenced peak milk yield and lactation persistency in the full dataset (P < 0.05; Fig. 1, Fig. 2). However, sensitivity analyses restricted to parities 1–4 showed that these interactions were no longer significant for peak milk yield (F = 1.660, *P* = 0.132) or lactation persistency (F = 0.496, *P* = 0.811), indicating that the interactions observed in the complete dataset were primarily driven by variation among fifth-parity cows.

### 3.2 Relationships among first-lactation traits and lifetime milk yield

Correlation coefficients among first-lactation production traits and estimated lifetime milk yield (ELMY) are presented in Table 3. Estimated lifetime milk yield was positively associated with first-lactation daily milk yield (*r* = 0.849; *P* < 0.01), peak milk yield (*r* = 0.706; *P* < 0.01), and lactation persistency (*r* = 0.458; *P* < 0.01). Among the evaluated predictors, daily milk yield exhibited the strongest relationship with ELMY. In contrast, lactation length showed no significant association with ELMY (*r* = 0.051; *P* > 0.05).

**Table 3:** Pearson correlation coefficients among first-lactation milk production traits and estimated lifetime milk yield in dairy cows.

	FPMY (kg)	FP (%)	FDMY (Kg)	FLL	ELMY
FPMY (kg)	1	0.211ns	0.889⁎⁎	0.045ns	0.706⁎⁎
FP (%)		1	0.509⁎⁎	0.235*	0.458⁎⁎
FDMY (kg)			1	0.021ns	0.849⁎⁎
FLL				1	0.051ns
ELMY					1

⁎⁎ : Correlation is significant at the 0.01 level;.

⁎ : Correlation is significant at the 0.05 level; ns: not significant; FLL: First-lactation length; ELMY: lifetime milk yield; FDMY: First-lactation daily milk yield; FPMY: First-lactation peak milk yield; FP: First-lactation persistency.

Among the candidate predictor variables, daily milk yield was strongly correlated with peak milk yield (*r* = 0.889; *P* < 0.01), whereas moderate positive associations were observed between persistency and daily milk yield (*r* = 0.509; *P* < 0.01) and between persistency and lactation length (*r* = 0.235; *P* < 0.05). The strong relationship between daily and peak milk yield suggested potential collinearity between these variables and warranted further assessment before their inclusion in regression models.

### 3.3 Prediction of lifetime milk yield

The results of regression analyses predicting the estimated lifetime milk yield based on the predictor variables, daily yield, and breed are shown in Tables 4a, b, c, Table 4d, Table 4e. Prior to model development, multicollinearity among candidate predictor variables was assessed using the tolerance statistic and variance inflation factors (VIF). First-lactation peak milk yield exhibited substantial collinearity with daily milk yield and was therefore excluded from subsequent regression analyses.

**Table 4a:** Summary statistics for multiple linear regression models predicting estimated lifetime milk yield from first-lactation milk production traits.

Model	Predictors	R	R²	Adjusted R²	RMSE
Model 1	FDMY	0.849	0.721	0.717	3592.33
Model 2	FDMY, Breed (Jersey (JER) and Crossbred (CRB))	0.863	0.744	0.735	3478.17

**Table 4b:** Overall regression analysis of variance (ANOVA) for Models 1 and 2 predicting estimated lifetime milk yield.

Model	df Regression	df Residual	F-value	P-value
Model 1	1	83	214.17	<0.001
Model 2	3	81	78.67	<0.001

**Table 4c:** Regression coefficients for Model 1 predicting estimated lifetime milk yield using first-lactation milk production traits.

Predictor	B	SE	β	t	P-value
Intercept	−5978.43	1751.23	-	−3.41	<0.001
FDMY	1836.56	125.50	0.849	14.63	<0.001

SE: standard error; B:.

**Table 4d:** Regression coefficients for the final prediction model (Model 2) of estimated lifetime milk yield based on first-lactation milk production traits and breed.

Predictor	B	SE	β	t	P-value
Intercept	−2003.18	2286.75	–	−0.88	0.384
FDMY	1651.03	142.72	0.763	11.59	<0.001
Jersey	−2387.173	1070.071	−0.162	−2.231	0.028
Crossbred	−2449.17	967.11	−0.168	−2.53	0.013

**Table 4e:** Collinearity diagnostics for predictor variables included in Models 1 and 2 for estimating lifetime milk yield.

Predictor	Tolerance	VIF	Tolerance	VIF
	Model 1		Model 2	
FDMY	1.000	1.000	0.725	1.380
Jersey (JER)	-	-	0.599	1.670
Crossbred (CRB)	-	-	0.717	1.395

Following variable screening, multiple regression analyses were performed to evaluate the ability of first-lactation production traits to predict estimated lifetime milk yield (ELMY). In the initial model, first-lactation daily milk yield (FDMY), persistency, and lactation length were included as explanatory variables. However, persistency and lactation length did not contribute significantly to model performance and were subsequently removed.

The final reduced model retained FDMY as the sole predictor of ELMY and demonstrated a strong positive relationship between first-lactation milk production and lifetime milk yield (Tables 4a, b). FDMY accounted for a substantial proportion of the variation in ELMY, indicating that cows producing more milk during their first lactation tended to achieve greater lifetime milk production.

To determine whether breed improved prediction accuracy, breed effects were incorporated into the regression model using Holstein as the reference category. The final model retained FDMY together with Jersey and Crossbred breed indicators. Including breed improved model performance, resulting in higher coefficients of determination and adjusted coefficients of determination compared with the model containing FDMY alone (Tables 4a, b). The final model explained approximately 74.4% of the variation in ELMY (Adjusted R² = 0.735; *P* < 0.001).

The regression equation describing the relationship between first-lactation milk yield and lifetime milk yield was:

ELMY=−2003.18+1651.03FDMY−2387.17JER−2449.17CRB

where ELMY represents estimated lifetime milk yield (kg), FDMY is first-lactation daily milk yield (kg/day), JER is the Jersey breed dummy variable (1 = Jersey, 0 = otherwise), and CRB is the Crossbred dummy variable (1 = Crossbred, 0 = otherwise).

The positive coefficient for FDMY indicates that increases in first-lactation milk yield were associated with higher lifetime milk production across breeds. Relative to Holstein cows, negative breed coefficients for Jersey and Crossbred cows indicated lower predicted lifetime milk production after accounting for differences in first-lactation milk yield.

To evaluate whether the relationship between first-lactation daily milk yield and estimated lifetime milk yield differed among breeds, interaction terms between FDMY and breed were fitted. Neither the FDMY × Jersey interaction (*P* = 0.569) nor the FDMY × Crossbred interaction (*P* = 0.746) was significant, indicating that the slope of the relationship between first-lactation milk yield and lifetime milk yield was similar across breeds. Consequently, a common prediction equation was considered appropriate for all breeds. Collectively, these results indicate that first-lactation daily milk yield is the primary determinant of lifetime milk yield in the study population and provides a robust basis for the early prediction of lifetime milk yield.

### 3.4 Breed-specific prediction models

To further investigate whether the predictive relationship between first-lactation milk yield and lifetime milk yield differed among breeds, separate regression models were fitted for Holstein, Jersey, and crossbred cows (Table 5). First-lactation daily milk yield was a significant predictor of estimated lifetime milk yield in all three breed groups (*P* < 0.001). However, the strength of the relationship varied among breeds. The highest predictive accuracy was observed in crossbred cows, where first-lactation daily milk yield explained 80.0% of the variation in estimated lifetime milk yield (Adjusted R² = 0.800).

**Table 5:** Breed-specific regression equations predicting estimated lifetime milk yield from first-lactation daily milk yield in Holstein, Jersey, and crossbred dairy cows.

Breed	Prediction equation	Adjusted R^2^	F-value	P-value
Holstein	*ELMY = −2188.47+1663.01(FDMY)*	0.494	33.26	<0.001
Jersey	*ELMY= −2061.35+1450.18 (FDMY)*	0.578	33.85	<0.001
Crossbred	*ELMY = −6019.06+1770.63 (FDMY)*	0.800	101.25	<0.001

In comparison, first-lactation daily milk yield explained 57.8% and 49.4% of the variation in estimated lifetime milk yield in Jersey and Holstein cows, respectively.

Regression coefficients also differed among breeds. A one-unit increase in first-lactation daily milk yield was associated with an increase of approximately 1663 kg, 1450 kg, and 1771 kg in estimated lifetime milk yield for Holstein, Jersey, and crossbred cows, respectively. Despite these numerical differences, subsequent interaction analysis revealed no significant breed × first-lactation milk yield interactions, indicating that the overall relationship between first-lactation performance and lifetime milk yield remained broadly consistent across breeds.

### 3.5 Classification of lifetime milk yield groups

To evaluate the practical relevance of first-lactation milk yield as an indicator of future performance, cows were classified into low-, medium-, and high-milk yield groups based on tertiles of estimated lifetime milk yield (Table 6). Significant differences in first-lactation daily milk yield were observed among milk yield classes (*F* = 84.78; *P* < 0.001). High lifetime producers exhibited the greatest first-lactation milk yield (17.03 ± 2.11 kg/day), followed by medium producers (13.79 ± 1.75 kg/day) and low producers (9.80 ± 1.57 kg/day). Pairwise comparisons revealed significant differences among all milk yield classes (*P* < 0.05).

**Table 6:** First-lactation daily milk yield (mean ± SE) across estimated lifetime milk-yield classes in dairy cows.

Milk yield class	Mean ELMY (kg)	FDMY (kg/day)
Low producer	10,734.33±1239.15	9.80±1.57^c^
Medium producer	18,575.98±3391.11	13.79±1.75^b^
High producer	28,166.72±2392.24	17.03±2.11^a^
ANOVA (F-value)		84.78
P-value		P < 0.001

abc Different superscripts within a column indicate significant differences according to Tukey's HSD test (*P* < 0.05). Milk yield classes were defined using tertiles of estimated lifetime milk yield (ELMY): FDMY: First-lactation daily milk yield.

The mean first-lactation milk yield of high producers was approximately 74% greater than that of low producers and 23% greater than that of medium producers. These findings indicate that cows that ultimately achieved superior lifetime milk yield could already be distinguished during their first lactation.

Binary logistic regression was used to evaluate whether first-lactation daily milk yield independently predicted classification into the high-estimated-lifetime-milk-yield group after adjustment for breed (Table 7). The overall model was statistically significant (Omnibus χ² = 47.600, df = 3, *P* < 0.001), indicating that the included predictors significantly improved the prediction of high estimated lifetime milk-yield status compared with the intercept-only model. The model demonstrated good explanatory performance (Nagelkerke pseudo-*R*² = 0.637) and an adequate fit to the data (Hosmer–Lemeshow χ² = 3.888, df = 7, *P* = 0.793).

**Table 7:** Binary logistic regression predicting high estimated lifetime milk-yield status from first-lactation daily milk yield after adjustment for breed.

Predictor	B	SE	Wald χ²	P-value	Odds ratio (95% CI)
First-lactation daily milk yield	0.760	0.200	14.438	<0.001	2.139 (1.445–3.167)
Jersey vs Holstein	−1.590	1.217	1.709	0.191	0.204 (0.019–2.212)
Crossbred vs Holstein	−1.424	0.947	2.262	0.133	0.241 (0.038–1.540)

Model χ² = 47.600, *P* < 0.001; Nagelkerke R² = 0.637; Hosmer–Lemeshow χ² = 3.888, *P* = 0.793; overall classification accuracy = 90.6%.

First-lactation daily milk yield was a significant independent predictor of high estimated lifetime milk-yield status (*B* = 0.760, SE = 0.200, Wald χ² = 14.438, *P* < 0.001). After adjustment for breed, each 1 kg/day increase in first-lactation daily milk yield increased the odds of belonging to the high estimated lifetime milk-yield group by approximately 2.14-fold (OR = 2.139, 95% CI: 1.445–3.167).

Breed was not independently associated with high estimated lifetime milk-yield status after accounting for first-lactation daily milk yield. Compared with Holstein cows, neither Jersey cows (OR = 0.204, 95% CI: 0.019–2.212, *P* = 0.191) nor Holstein × Jersey crossbred cows (OR = 0.241, 95% CI: 0.038–1.540, *P* = 0.133) differed significantly in their odds of being classified as high lifetime producers.

The final model correctly classified 90.6% of cows, with a specificity of 95.3% for identifying non-high producers and a sensitivity of 76.2% for identifying high producers.

### 3.6 Identification of a first-lactation milk yield threshold for replacement decisions

**Fig. 3:**
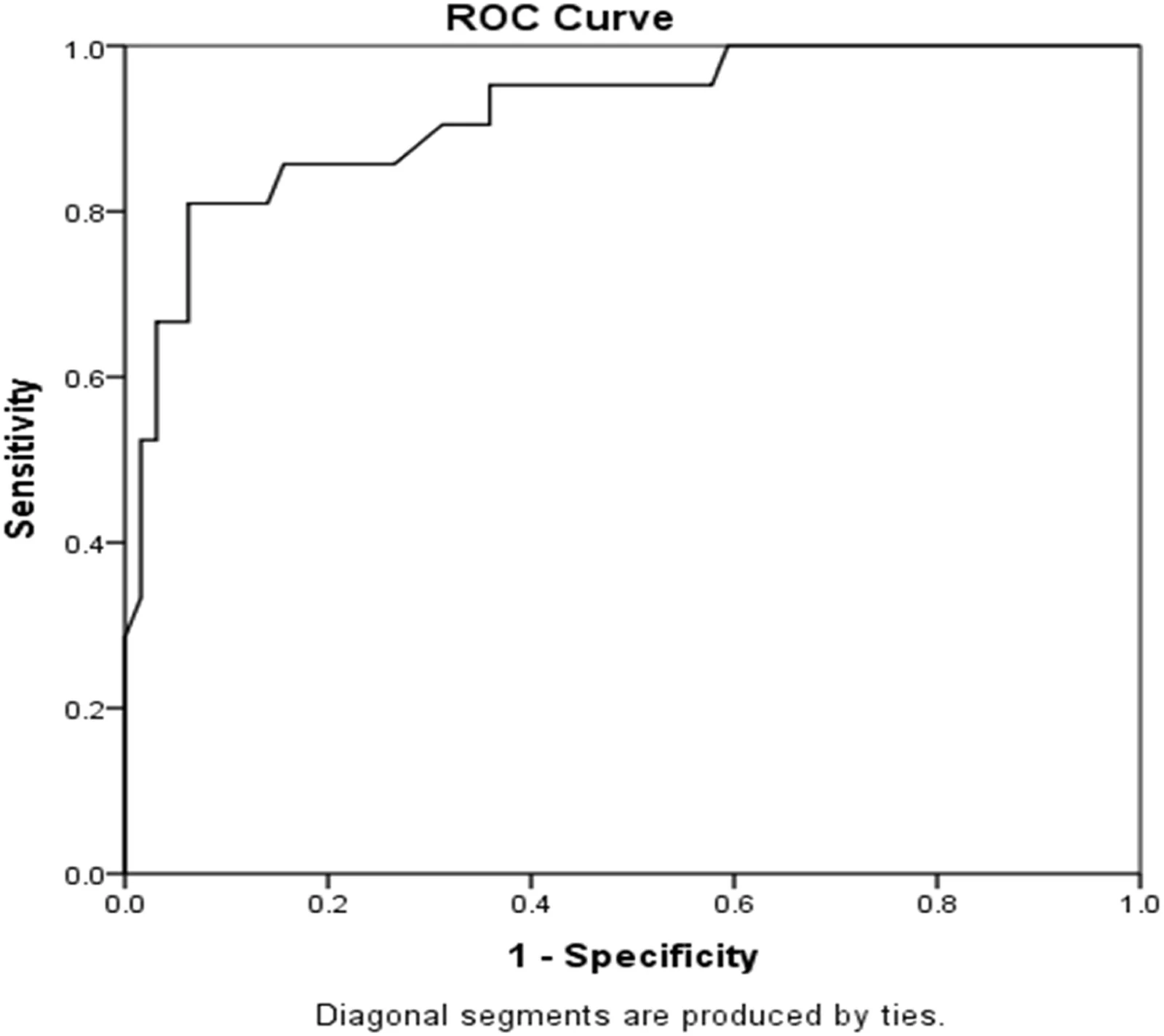
Receiver operating characteristic (ROC) curve describing the ability of first-lactation daily milk yield to discriminate high lifetime-producing cows from low- and medium-producing cows.

Given the strong predictive performance of first-lactation daily milk yield in the logistic regression model, receiver operating characteristic (ROC) analysis was subsequently performed to evaluate its discriminatory ability and identify an optimal threshold for distinguishing high-estimated-lifetime milk producers (Fig. 3; Table 8).

**Table 8:** Receiver operating characteristic (ROC) analysis evaluating the diagnostic performance of first-lactation daily milk yield for identifying high estimated lifetime milk-producing dairy cows.

Area under the curve (AUC)	Standard error	P-value	Optimal threshold (kg/day)	Sensitivity (%)	Specificity (%)
0.917 (95% CI: 0.846–0.988)	0.036	<0.001	16.0	76.2	93.7

Optimal threshold determined using the maximum Youden Index.

First-lactation daily milk yield demonstrated excellent discriminatory ability for identifying cows that subsequently achieved high lifetime milk yield, with an area under the ROC curve (AUC) of 0.917 ± 0.036 (*P* < 0.001). This indicates a high probability that a randomly selected high-producing cow would have a greater first-lactation milk yield than a randomly selected low- or medium-producing cow.

The optimal classification threshold was identified at approximately 16.0 kg/day. At this threshold, sensitivity was 76.2%, indicating that more than three-quarters of future high-producing cows were correctly identified during first lactation. Specificity was 93.7%, demonstrating a high capacity to correctly exclude cows that did not subsequently achieve high lifetime milk yield.

These results suggest that first-lactation milk yield can be used as a practical indicator for early replacement and retention decisions. Cows producing at least 16 kg/day during first lactation were substantially more likely to become high lifetime producers than cows producing below this threshold.

## 4 Discussion

Milk production efficiency in dairy cattle is determined by the interaction of genetic potential, physiological maturity, and management conditions. The present study evaluated the effects of breed and parity on key milk production traits and subsequently examined the extent to which first-lactation performance could predict lifetime milk yield under subtropical production conditions. Across all analyses, first-lactation daily milk yield emerged as the most consistent indicator of future milk yield, highlighting its potential utility as a practical tool for selection and replacement.

The significant breed differences observed for daily milk yield, peak milk yield, lactation persistency, and lactation yield confirm the important role of genetic background in determining dairy cow milk yield. Holstein cows consistently outperformed Jersey and Crossbred cows for milk yield-related traits, whereas Jersey cows exhibited the lowest production levels. These findings agree with numerous studies demonstrating the superior milk-producing capacity of Holstein cattle owing to decades of intensive selection for increased milk yield and mammary secretory capacity (Capper & Cady, 2020; Olthof et al., 2023). Holsteins have undergone sustained genetic improvement for milk volume, resulting in enhanced nutrient partitioning towards lactation and greater metabolic capacity to support milk synthesis (Douglas et al., 2016; Davis et al., 2023).

Crossbred cows generally displayed intermediate performance between Holstein and Jersey cows. This observation supports previous reports indicating that crossbreeding can combine desirable characteristics of parental breeds through heterosis, resulting in improved milk yield relative to lower-yielding breeds while retaining adaptive and functional advantages often absent in highly specialised dairy genotypes (Clasen et al., 2017; Ferris et al., 2018). Although crossbreds did not match Holsteins for milk yield, their intermediate performance suggests that crossbreeding remains a viable strategy for balancing milk yield and adaptability under variable production environments. The lower milk yields observed in Jersey cows are consistent with their recognised biological specialisation for milk solids concentration, feed efficiency, and reproductive performance rather than milk volume alone (Capper & Cady, 2020; Olthof et al., 2023).

Parity exerted a significant influence on all milk production traits except lactation length, with milk yield-related traits generally increasing from first to later parities. This pattern reflects the well-established process of physiological maturation in dairy cows. As cows progress through successive lactations, increases in body size, digestive capacity, endocrine responsiveness, and mammary gland development enhance their capacity for milk production (Xiong et al., 2018; Lacková et al., 2022; Davis et al., 2023). The progressive increase in daily milk yield, peak milk yield, and lactation yield from first through fourth and fifth parity observed in the present study is therefore consistent with biological expectations. Similar parity-associated increases in milk production have been reported across a range of dairy production systems worldwide (Vrhel et al., 2024).

In contrast, lactation length remained relatively stable across breeds and parities. Because neither breed nor parity significantly influenced lactation length in the present study, the observed consistency may reflect the relatively uniform herd management and reproductive scheduling under which the cows were maintained. Similar observations have been reported in intensively managed dairy systems, where lactation length is often influenced by breeding targets and calving intervals rather than production characteristics alone (Williams et al., 2021). Consequently, variation in lactation length may be less responsive to breed and parity effects than yield-related traits.

Although significant breed × parity interactions were detected for peak milk yield and lactation persistency in the complete dataset, sensitivity analyses restricted to parities 1–4 showed that these interactions were no longer significant. This suggests that breed differences were generally stable during the first four lactations, whereas the interactions observed in the full dataset primarily reflected breed divergence in the fifth parity. Such divergence may indicate age-related differences in productive longevity, physiological resilience, and maturity rates among cows that remained in production beyond the fourth lactation rather than fundamental changes in genetic superiority (Shokri-Sangari et al., 2019; Arianfar et al., 2022; Barua et al., 2022).

The correlation analysis provided important insight into the relationships among first-lactation production traits and lifetime milk yield. First-lactation daily milk yield exhibited the strongest association with estimated lifetime milk yield, supporting the hypothesis that early-life production performance reflects future productive potential. Peak milk yield was also strongly correlated with lifetime milk yield; however, its exceptionally high correlation with daily milk yield indicated substantial redundancy between the two traits. Similar relationships have been reported in dairy cattle populations, in which peak and average milk yields capture overlapping aspects of lactational performance (Mellado et al., 2011a, Mellado et al., 2011b; Erdoğan et al., 2024). Consequently, exclusion of peak milk yield from subsequent predictive models was statistically justified and improved model stability by reducing multicollinearity.

Although lactation persistency was positively associated with lifetime milk yield, its relationship was considerably weaker than that observed for daily milk yield. Persistency has long been recognised as an economically important trait because it influences energy balance, feed efficiency, health status, and the shape of the lactation curve (Erdoğan et al., 2024). Nevertheless, the moderate correlations observed in the present study suggest that persistency contributes indirectly to lifetime milk yield and may be overshadowed by the dominant influence of milk production level itself. Similarly, the lack of a significant relationship between lactation length and estimated lifetime milk yield may reflect the relatively uniform management conditions under which cows were maintained. In intensive production systems, differences in milk yield are often more influential determinants of cumulative production than variation in lactation duration alone (Gaillard et al., 2016).

The regression analyses further demonstrated the importance of first-lactation daily milk yield as a predictor of estimated lifetime milk yield. The relatively high coefficient of determination of the final prediction models indicates that first-lactation performance accounted for a substantial proportion of the variation in estimated lifetime milk yield, consistent with previous evidence that early-lactation records can provide useful information on subsequent production performance, longevity, and economic merit (Jenko et al., 2015; Siriak et al., 2022; Dallago et al., 2024). Several interrelated biological mechanisms may underpin this predictive relationship, including mammary developmental capacity, metabolic adaptation, genetic merit, and physiological resilience. Mammary development during the first lactation contributes to the functional secretory capacity of the udder, with greater development and numbers of active secretory cells supporting higher milk production in subsequent lactations (Murney et al., 2015). Similarly, cows capable of sustaining relatively high milk production during the first lactation may possess greater capacity to adapt metabolically to the energetic demands of lactation, facilitating effective nutrient partitioning towards milk synthesis while maintaining physiological homeostasis (Ehrhardt et al., 2016; Humer et al., 2016; Favorit et al., 2021). First-lactation performance may also partly reflect underlying genetic merit for milk production, as cows with greater genetic potential tend to maintain superior production across successive lactations (Kasiviswanathan et al., 2024). Finally, the capacity to sustain production across multiple lactations may reflect greater biological resilience and an enhanced ability to cope with nutritional, metabolic, and environmental challenges (Punturiero et al., 2025; Agyiri et al., 2026).

Incorporating breed into the prediction model resulted in a modest improvement in predictive performance, indicating that breed provided additional information beyond first-lactation daily milk yield alone. After adjustment for first-lactation performance, predicted ELMY differed among breeds, with Holstein cows having higher predicted values than Jersey and Crossbred cows. This finding indicates that breed-specific differences persisted even after accounting for early milk production. However, the relatively small improvement in model performance after including breed suggests that first-lactation daily milk yield accounted for a considerably greater proportion of the variation in ELMY than breed alone did.

Breed-specific regression analyses further showed that the strength of the relationship between first-lactation daily milk yield and ELMY varied among breeds. The strongest relationship was observed in Crossbred cows, for which first-lactation daily milk yield explained approximately 80% of the variation in ELMY. Crossbreeding has been associated with improved functional efficiency, environmental adaptability, and resilience (Clasen et al., 2017; Ferris et al., 2018), which may partly contribute to the stronger relationship observed in Crossbred cows. However, the breed × first-lactation daily milk yield interaction was not significant, indicating no statistical evidence that the association between first-lactation daily milk yield and ELMY differed among breeds. Thus, while the predictive strength varied numerically among breed-specific models, the overall relationship between early milk production and ELMY was generally consistent across the three genetic groups.

An important extension of the predictive analyses was the classification of cows according to ELMY, which provided an opportunity to evaluate whether first-lactation performance could discriminate among cows with differing subsequent production outcomes. Cows classified as high ELMY producers had significantly greater first-lactation daily milk yields than those in the medium- and low-ELMY groups, suggesting that differences in longer-term milk production potential may already be detectable during the first lactation. This pattern is consistent with previous studies reporting associations between superior first-lactation performance and greater cumulative milk production, longevity, and lifetime profitability (Siriak et al., 2022; Dallago et al., 2024).

The binary logistic regression analysis further demonstrated that first-lactation daily milk yield was significantly associated with the likelihood of classification into the high-ELMY group, independent of breed. Specifically, each 1 kg/day increase in first-lactation daily milk yield was associated with approximately a two-fold increase in the odds of belonging to the high-ELMY group. This result complemented the linear regression findings by demonstrating that first-lactation daily milk yield was informative not only for predicting continuous ELMY but also for distinguishing cows with a greater likelihood of achieving high ELMY.

After adjustment for first-lactation daily milk yield, neither Jersey nor Holstein × Jersey crossbred cows differed significantly from Holstein cows in the likelihood of belonging to the high-ELMY group. This finding further indicates that, within the present population, first-lactation daily milk yield provided greater discriminatory information for high ELMY status than breed. However, the relatively small number of high-ELMY cows and the wide confidence intervals around the breed estimates indicate limited precision; therefore, the absence of significant breed effects should not be interpreted as evidence of equivalence among breeds. Larger, independent populations are needed to determine whether breed contributes additional predictive information beyond first-lactation milk yield.

The ROC analysis further demonstrated the potential utility of first-lactation daily milk yield for discriminating cows according to subsequent ELMY status. The high area under the curve (AUC = 0.917) indicated strong discrimination within the study population, with values above 0.90 generally considered indicative of outstanding classification performance (Mandrekar, 2010). An optimal threshold of approximately 16 kg/day provided a favourable balance between sensitivity and specificity, suggesting its potential value as an early decision-support indicator for identifying cows more likely to belong to the high-ELMY group. However, because the ROC-derived threshold was established from the same single-herd retrospective dataset used for model development and was not subjected to internal or external validation, its performance may be population-specific. Accordingly, the 16 kg/day threshold should be regarded as preliminary, and validation in independent dairy populations is required before its broader application in herd management.

The identification of a data-derived threshold extends the analysis beyond statistical association by providing a potentially interpretable benchmark for herd-level decision support. Although previous studies have reported associations between early-lactation performance and lifetime milk yield (Jenko et al., 2015; Siriak et al., 2022), operational thresholds for identifying cows with greater long-term milk production potential under commercial conditions remain limited. In the present population, cows producing at least 16 kg/day during their first lactation were more likely to be classified as high-ELMY. This preliminary threshold may therefore complement existing approaches for early evaluation of cows, particularly under comparable subtropical production conditions. However, it should not be regarded as a stand-alone criterion for culling or replacement; rather, first-lactation milk yield should be considered alongside reproductive performance, udder health, lameness, genetic merit, temperament, expected longevity, and the economic implications of retaining or replacing individual cows.

This study has some limitations that should be considered. First, lifetime milk yield was estimated rather than recorded directly because complete cumulative lactation-yield records were unavailable in the historical farm database. Lactation yields were derived consistently from average daily milk yield and lactation length across consecutive lactations, providing a standardised estimate of cumulative milk production for all cows. Although this approach may not capture all within-lactation fluctuations in milk yield, it enabled consistent longitudinal comparisons among animals using the available records. The resulting prediction models and threshold should therefore be interpreted in relation to estimated lifetime milk yield (ELMY) within the study population. Second, the study was based on production records from a single commercial dairy herd managed under relatively uniform environmental and management conditions. While this provided consistency in the production environment, the prediction models and proposed first-lactation milk-yield threshold have not been externally validated and should therefore be regarded as preliminary and most relevant to herds managed under comparable subtropical production conditions. Third, the requirement for at least three consecutive lactation records provided sufficient longitudinal information for estimating cumulative milk yield but excluded cows removed during the first or second lactation because of production, reproductive, health, or other factors. This restriction may introduce some degree of survivorship bias and should be considered when extrapolating the predictive findings to cows at risk of early removal. Fourth, potentially important determinants of cumulative milk production, including age at first calving, year and season of calving, calving interval, reproductive performance, disease occurrence, genetic merit, and reasons for culling, were either unavailable in the historical records and, therefore, could not be incorporated into the models. Their omission may have contributed to residual confounding. Future studies incorporating larger, independent multi-herd populations, cows removed during early lactation, more comprehensive health and reproductive information, and directly recorded cumulative lactation yields or detailed test-day yields would be valuable for validating and refining the prediction models and the proposed threshold.

## 5 Conclusion

This study demonstrated significant effects of breed and parity on milk production traits in dairy cattle managed under subtropical conditions. Holstein cows generally had higher daily, peak, and lactation milk yields than Jersey and Holstein × Jersey crossbred cows, while milk production tended to increase with advancing parity. Although breed × parity interactions were evident for selected traits, the overall patterns of breed differences were largely maintained across successive lactations.

Among the first-lactation traits evaluated, daily milk yield showed the strongest predictive relationship with estimated lifetime milk yield (ELMY), with this association generally maintained across the three genetic groups. Complementary logistic regression and ROC analyses further indicated that first-lactation daily milk yield could discriminate cows according to subsequent ELMY status within the study population. A threshold of approximately 16 kg/day provided a favourable balance between sensitivity and specificity for identifying cows in the high-ELMY group.

The identified threshold should, however, be regarded as a preliminary decision-support indicator rather than a stand-alone criterion for replacement or selection decisions. Its interpretation should be confined primarily to production conditions comparable to those represented in this study and considered alongside other health, reproductive, genetic, and economic factors relevant to herd management. Given the retrospective single-herd design, use of estimated lifetime milk yield, and restriction of the prediction dataset to cows with at least three consecutive lactations, validation in larger, independent multi-herd populations is required before broader application.

## Statement for studies in humans and animals

This study was conducted using retrospective production records obtained from a Dairy Farm, in the Eastern Cape, South Africa. No live animals were handled, sampled, restrained, treated, or subjected to any experimental procedures for the purpose of this research. The study involved the analysis of existing farm records only and therefore did not require direct animal involvement.

The data used in this study were routinely collected by the farm as part of normal herd management activities prior to the commencement of the research. Consequently, no additional procedures were performed on animals, and animal welfare was not affected by the study.

The authors confirm that the research was conducted in accordance with institutional policies governing the use of farm production records for research purposes.

## Funding

No external funding was received for this study.

## CRediT authorship contribution statement

**Onke Tshona:** Investigation, Writing – original draft. **Ziyanda Mpetile:** Conceptualization, Data curation, Resources, Writing – review & editing, Supervision. **Oluwakamisi Festus Akinmoladun:** Conceptualization, Data curation, Visualization, Validation, Software, Methodology, Formal analysis, Writing – review & editing, Supervision.

## Declaration of competing interest

The authors declare that they have no known competing financial interests or personal relationships that could have influenced the work reported in this paper.

## References

[bib0002] Agyiri M., Onzima R., Costa A., Fuerst-Waltl B., Steininger F., Egger-Danner C., Sölkner J. (2026). Analysing resilience indicator traits in Austrian Fleckvieh, Brown swiss and Holstein-Friesian cattle. Journal of Animal Breeding and Genetics.

[bib0004] Arianfar M., Rokouei M., Dashab G.R., Faraji-Arough H. (2022). Investigating the relationship between lactation curve parameters and some economic traits of Iranian Holstein cows. Animal Production Research.

[bib0005] Barua K., Akter N., Alam M., Bari M.S., Sultan M.N., Islam S., Hossain M.E. (2022). Effects of genotype, parity, season and their interactions on milk yield in crossbred dairy cattle. Journal of Animal Physiology and Animal Nutrition.

[bib0006] Bieber A., Hinrichs D., Moser F.N., Maeschli A., Lora I., Cozzi G., Leiber F. (2026). Predicting longevity-related traits in Swiss low-input and organic dairy cows from herdbook data of first versus second lactation. Veterinary and Animal Science.

[bib0008] Capper J.L., Cady R.A. (2020). The effects of improved performance in the US dairy cattle industry on environmental impacts between 2007 and 2017. Journal of Animal Science.

[bib0009] Clasen J.B., Fikse W.F., Ramin M., Lindberg M. (2024). Effects of herd management decisions on dairy cow longevity, farm profitability, and emissions of enteric methane–a simulation study of milk and beef production. Animal : An international journal of animal bioscience.

[bib0010] Clasen J.B., Norberg E., Madsen P., Pedersen J., Kargo M. (2017). Estimation of genetic parameters and heterosis for longevity in crossbred Danish dairy cattle. Journal of Dairy Science.

[bib0011] Dallago G.M., Elsohaby I., McClure J.T., Lacroix R., Vasseur E. (2024). The associations of early-life health and performance with subsequent dairy cow longevity, milk yield, and profitability. Animal : An International Journal of Animal Bioscience.

[bib0012] Davis L., French E.A., Aguerre M.J., Ali A. (2023). Impact of parity on cow stress, behavior, and production at a farm with guided traffic automatic milking system. Frontiers in Animal Science.

[bib0013] Davis S.R. (2017). Triennial lactation symposium/bolfa: Mammary growth during pregnancy and lactation and its relationship with milk yield. Journal of Animal Science.

[bib0014] De Vries A., Marcondes M.I. (2020). Overview of factors affecting productive lifespan of dairy cows. Animal : An International Journal of Animal Bioscience.

[bib0015] Douglas M.L., Marett L.C., Macmillan K.L., Morton J.M., Hannah M.C., Fisher A.D., Auldist M.J. (2016). Associations of high and low milk protein concentrations with energy allocation, milk production, and concentrations of blood plasma metabolites and hormones in Holstein-Friesian cows. Journal of Dairy Science.

[bib0016] Ehrhardt R.A., Foskolos A., Giesy S.L., Wesolowski S.R., Krumm C.S., Butler W.R., Boisclair Y.R. (2016). Increased plasma leptin attenuates adaptive metabolism in early lactating dairy cows. Journal of Endocrinology.

[bib0018] Erdoğan M., Çinkaya S., Brenig B., Çelikeloğlu K., Demirtaş M., Sarıibrahimoğlu S., Tekerli M. (2024). Genome-wide association studies for milk production traits and persistency of first calving Holstein cattle in Türkiye. Frontiers in Veterinary Science.

[bib0019] Favorit V., Hood W.R., Kavazis A.N., Skibiel A.L. (2021). Graduate student Literature Review: Mitochondrial adaptations across lactation and their molecular regulation in dairy cattle. Journal of Dairy Science.

[bib0020] Ferris C.P., Purcell P.J., Gordon A.W., Larsen T., Vestergaard M. (2018). Performance of Holstein and Swedish-Red× Jersey/Holstein crossbred dairy cows within low-and medium-concentrate grassland-based systems. Journal of Dairy Science.

[bib0021] Gaillard C., Friggens N.C., Taghipoor M., Weisbjerg M.R., Lehmann J.O., Sehested J. (2016). Effects of an individual weight-adjusted feeding strategy in early lactation on milk production of Holstein cows during extended lactation. Journal of Dairy Science.

[bib0023] Humer E., Khol-Parisini A., Gruber L., Wittek T., Aschenbach J.R., Zebeli Q. (2016). Metabolic adaptation and reticuloruminal pH in periparturient dairy cows experiencing different lipolysis early postpartum. Animal : An International Journal of Animal Bioscience.

[bib0024] Jenko J., Perpar T., Kovač M. (2015). Genetic relationship between the lifetime milk production, longevity and first lactation milk yield in Slovenian Brown cattle breed. Mljekarstvo: Časopis za Unaprjeđenje Proizvodnje i Prerade Mlijeka.

[bib0025] Kasiviswanathan D., Devendran P., Venkataramanan R., Meenakshisundaram S., Senthilkumar G., Peters S.O. (2024). Genetic evaluation of monthly test-day milk yields of Jersey crossbred cattle under farmers’ Production system in Tamil Nadu, India. Animals.

[bib0028] Lacková P.T., Maskaľová I., Vajda V. (2022). Effect of parity and days in milk on milk urea concentration and milk components in Holstein dairy cows. Folia Veterinaria.

[bib43] Mandrekar J.N. (2010). Receiver operating characteristic curve in diagnostic test assessment. Journal of Thoracic Oncology.

[bib0029] Mellado M., Antonio-Chirino E., Meza-Herrera C., Veliz F.G., Arevalo J.R., Mellado J., De Santiago A. (2011). Effect of lactation number, year, and season of initiation of lactation on milk yield of cows hormonally induced into lactation and treated with recombinant bovine somatotropin. Journal of Dairy Science.

[bib0030] Mellado M., Coronel F., Estrada A., Ríos F.G. (2011). Lactation performance of Holstein and Holstein x Gyr cattle under intensive condition in a subtropical environment. Tropical and Subtropical Agroecosystems.

[bib0032] Murney R., Stelwagen K., Wheeler T.T., Margerison J.K., Singh K. (2015). The effects of milking frequency in early lactation on milk yield, mammary cell turnover, and secretory activity in grazing dairy cows. Journal of Dairy Science.

[bib0033] Olthof L.A., Domecq J.J., Bradford B.J. (2023). Analysis of Jersey versus Holstein breed profitability on north central US dairies. JDS Communications.

[bib0034] Punturiero C., Delledonne A., Ferrari C., Bagnato A., Strillacci M.G. (2025). Mapping genomic regions affecting resilience traits in a large dairy farm of Holstein cows. Frontiers in Animal Science.

[bib0035] Schlebusch S., Hoop D., von Rohr P., Pausch H., Gazzarin C. (2024). Enhancing culling decisions in Swiss dairy farming: Introducing a tool for improved replacement choices. Smart Agricultural Technology.

[bib0036] Shokri-Sangari F., Atashi H., Dadpasand M., Saghanejad F. (2019). Genetic parameters for milk yield and lactation persistency in the three first parities of Iranian Holstein cows. Revista Colombiana de Ciencias Pecuarias.

[bib0037] V. Siriak, Y. Polupan, & R. Stavetska (2022). Retrospective: Duration and efficiency of dairy cows productive lifespan depending on age at first calving and first lactation milk yield.

[bib0040] X. Tong, T. X., X.J Wang, W. X., D.G. Li, L. D., H.Q Zhu, Z. H., D.Y. Ding, D. D., L. Min, M. L., … & G. Wang, W. G. (2018). Variation in milk performance of Holstein dairy cows under the effects of season, parity and lactation stage in South China.

[bib0041] Vrhel M., Ducháček J., Gašparík M., Vacek M., Codl R., Pytlík J. (2024). Association between production and reproduction parameters based on parity and breed of dairy cows in the Czech Republic. Archives Animal Breeding.

[bib0042] Williams M., Murphy C.P., Sleator R.D., Ring S.C., Berry D.P. (2021). Genetic and nongenetic factors associated with lactation length in seasonal-calving, pasture-based dairy cows. Journal of Dairy Science.

